# Early postnatal demoralisation among primiparous women in the community: measurement, prevalence and associated factors

**DOI:** 10.1186/s12884-015-0680-3

**Published:** 2015-10-12

**Authors:** Irene Bobevski, Heather Rowe, David M. Clarke, Dean P. McKenzie, Jane Fisher

**Affiliations:** 1 Jean Hailes Research Unit, School of Public Health and Preventive Medicine, Monash University, Melbourne, Australia; 2 Department of Psychiatry, School of Clinical Sciences at Monash Health, Faculty of Medicine, Nursing and Health Sciences, Monash University, Melbourne, Australia; 3 Department of Epidemiology and Preventive Medicine, School of Public Health and Preventive Medicine, Monash University, Melbourne, Australia; 4 Research Development & Governance, Epworth HealthCare, Melbourne, Australia

**Keywords:** Demoralisation, Postnatal mental health, Early parenting

## Abstract

**Background:**

Demoralisation is a psychological state occurring in stressful life situations where a person feels unable to respond effectively to their circumstances, characterised by feelings of distress, subjective incompetence, helplessness and hopelessness. The period after the birth of a first baby is a time of great changes and disruptions to many aspects of the mother's physical, psychological and social functioning. This can lead to feelings of distress, a sense of incompetence and helplessness. This study aimed to examine: (1) the psychometric properties of the Demoralisation Scale in a community setting; (2) the prevalence of demoralisation symptoms among primiparous women in the community; and (3) factors that are uniquely associated with demoralisation in the early postnatal period.

**Methods:**

Primiparous women attending community maternal health centres (*n* = 400) were recruited and administered the study's questionnaires through a telephone interview.

**Results:**

The Demoralisation Scale was found to be a reliable and valid tool among women in the community who had recently given birth. Higher levels of demoralisation were independently associated with lower confidence on going home from the hospital after birth, lower rating of mother's self-rated global health, more than 3 h of infant crying and fussing in the last 24 h, and a controlling partner, after symptoms of depression and anxiety, and vulnerable personality characteristics were controlled for.

**Conclusions:**

The relevance of demoralisation to postnatal health practitioners in the community is in helping them to better understand women's experiences and to intervene in a way that is more meaningful and less stigmatising to women.

## Background

Demoralisation is a psychological state occurring in stressful life situations where a person feels that they are unable to respond effectively to their circumstances. It is characterised by feelings of distress, helplessness, subjective incompetence, and hopelessness [1, 2]. Demoralisation has been found to be distinct from the diminished ability to experience pleasure (anhedonia) that is an important hallmark of depression with melancholic features [3–7]. Demoralisation is generally viewed as a dimensional phenomenon that is not part of a psychiatric diagnostic system, despite some overlap with the Diagnostic and Statistical Manual of Mental Disorders, Fifth Edition (DSM-5) [8] criteria for Major Depressive Disorder. Subjective incompetence, which is a major characteristic of demoralisation is not adequately accounted for by existing diagnostic systems and dimensional measures of depression [9, 10].

So far demoralisation has predominantly been studied in people with physical illness (e.g. [11]). The only study of demoralisation in the postnatal period [12] examined this construct among Australian women with unsettled infants admitted to a hospital mother-baby unit for a five-day intervention program. This study found that a scale measuring demoralisation, the Demoralisation Scale [13], was a reliable, valid, and acceptable instrument in the above postnatal setting. Approximately half of the women experienced high levels of demoralisation, associated with more impaired functioning and negative experiences of motherhood (such as interaction and closeness to the baby, confidence as a mother, and feeling isolated and unsupported), independent of depression and anxiety symptoms. The intervention program, which focused on psycho-education and skill building, resulted in more participants improving significantly on demoralisation than on depression and anxiety symptoms.

The construct of demoralisation may be particularly relevant after the birth of a first baby, which is a time of great changes and disruptions to many aspects of the mother's physical, social and psychological functioning [14]. However, such experiences are often unrecognised and unnamed [15], usually in the context of community expectations that this should be an exclusively joyous period [16] and that the skills of caring for an infant are intuitive [17].

Lumley [18] pointed out that in order for population based mental health programs to be effective, it is important for health professionals to understand and acknowledge women's experiences in a way that women can relate to and perceive as meaningful and non-stigmatising. A qualitative study of women diagnosed with probable depression found that they believed that the most salient contributing factors were the context of their life as a mother, including lack of support, isolation, fatigue and physical ill health; having no time or space for the self. As many of these women did not believe their depression to be caused directly by the baby, they did not perceive their experiences as "postnatal depression" and therefore did not seek treatment [19]. The construct of demoralisation emphasises the context of the person's experiences, such as a first time mother responding to a demanding and stressful situation, in a way that current diagnostic systems of depression may not [9]. In addition to the traditional assessment of depression and anxiety, a valid measure of demoralisation that can be used in community settings may capture better the response of first time mothers to the demands of looking after a young infant in a more meaningful and less stigmatising way. Demoralisation also has relevance to population based postnatal programs in directing them towards helping first time mothers to acquire the necessary caregiving skills and to increase their parental self-efficacy, thereby decreasing their sense of helplessness and subjective incompetence [12].

The prevalence of demoralisation and its association with other postnatal factors, such as infant behaviour, parental confidence, breast feeding, mother's health, and the quality of relationship with partner during the early postnatal period have so far not been examined among women in the general community. Furthermore, personality characteristics, such as a tendency to worry, lack of assertiveness, interpersonal sensitivity, and harm avoidance have been consistently found to be risk factors for the development of postnatal depression [20–23]. It is also important to determine whether there is an association between demoralisation and personality characteristics, and whether demoralisation is associated with the above postnatal factors independently of personality traits and symptoms of depression and anxiety.

The aims of the present study were to examine: (1) the psychometric properties of the Demoralisation Scale in a community setting; (2) the prevalence of demoralisation symptoms among first time mothers in the community; and (3) factors that are uniquely associated with demoralisation in the early weeks after birth, including demographics, mode of delivery, mother's health, breast feeding, infant behaviour and relationship with partner.

## Methods

### Ethical approval

Ethical approval was obtained from the Southern Health (now Monash Health) Human Research Ethics Committee (24 April 2013; 11388B), the Monash University Human Research Ethics Committee (30 April 2013; CF12/1022-2012000474), and the Education and Policy Research Committee, Victorian Government Department of Education and Early Childhood Development (22 March 2012; 2012_001472).

### Study design

This study was nested within and employed baseline data from a cluster randomised controlled trial of a brief couple-focused psychoeducational intervention to prevent postnatal disorders among women, the What Were We Thinking (WWWT) programme. The study protocol is described in full elsewhere [24].

### Setting and sample

After women are discharged from hospital following birth primary postnatal health care in Victoria, Australia, is provided by Maternal and Child Health nurses in local Maternal and Child Health Centres (MCHCs), administered in Local Government Areas (LGA). This is a free government service accessible to all families. Participants were primiparous women who had recently given birth in the prior four weeks and were receiving postpartum care in a participating MCHC. Four hundred participants were recruited from 48 MCHCs selected from six LGAs, including high, medium, and low socioeconomic indexed areas [24]. Women whose English language proficiency was sufficient to give consent and complete structured telephone interviews were eligible to participate.

### Procedure

In the first instance women were contacted by an LGA officer who explained the study and asked those women who were interested for permission to pass their contact details to the research team. Within a week a member of the research team telephoned women who had provided contact details to invite them to participate in the study. A plain language explanation of the study and a consent form were mailed or emailed to women who expressed interest. A computer-assisted telephone interview (CATI) was conducted with women who gave consent. This formed the baseline data of the cluster randomised control study described above. Data for the present study was from the baseline interview, which occurred prior to the WWWT intervention being carried out. Participants and interviewers were blind as to whether participants were assigned to the intervention or control group.

### Measures

Study-specific questions were used to obtain information about participant's age, infant's age, education, marital status, language spoken at home, whether the pregnancy was unexpected, mode of birth (i.e. spontaneous vaginal, assisted vaginal, caesarean without labour, caesarean after a period of labour), parental confidence on leaving the hospital after birth. Parental confidence was assessed using a single question: "How confident did you feel on going home from the hospital?". Response options were on a scale from 1 "not at all" to 5 "very confident".

Demoralisation was measured with the Demoralisation Scale [13], consisting of 24 items (listed in Table 1), scored on a 5-point Likert scale, from 0 "Never" to 4 "All the Time". Total scores range from 0 to 96. Scale items are listed in Table 1. The scale was initially developed and validated with patients in palliative care [13], but has also been validated with women who had recently given birth in a hospital mother-baby unit [12].

**Table 1:** Item characteristics and exploratory factor analysis of the Demoralisation Scale

Factor (% of total variance explained)	Item mean (SD)	Corrected item-total correlation	Factor loadings			
			F1	F2	F3	F4
F1: Helplessness (35.4 %) (α = .83)						
9. I feel hopeless.	0.4 (0.7)	.64	.73			
7. No one can help me.	0.4 (0.8)	.52	.72			
8. I feel that I cannot help myself.	0.4 (0.7)	.62	.70			
22. I feel discouraged about life.	0.3 (0.6)	.68	.64			
10. I feel guilty.	0.7 (1.0)	.54	.58			
21. I feel sad and miserable.	0.7 (0.8)	.67	.51	.47		
F2: Disheartenment (7.2 %) (α = .79)						
4. My role in life has been lost.	0.4 (0.7)	.42		.76		
24. I feel trapped by what is happening to me.	0.4 (0.8)	.65		.68		
23. I feel quite isolated or alone.	0.8 (1.0)	.59		.58		
5. I no longer feel emotionally in control.	0.7 (0.9)	.54		.54		
18. I feel distressed about what is happening to me.	0.5 (0.8)	.65	.45	.51		
6. I am in good spirits.	0.9 (0.7)	.46		.43	.48	
F3: Sense of Failure (6.5 %) (α = .63)						
19. I am a worthwhile person.	0.7 (0.8)	.36			.73	
17. I am proud of my accomplishments.	0.8 (0.7)	.47			.67	
1. There is a lot of value in what I can offer others.	1.5 (0.8)	.27			.65	
12. I cope fairly well with life.	0.8 (0.7)	.49			.49	
F4: Dysphoria (5.0 %) (α = .71)						
15. I tend to feel hurt easily.	1.1 (0.9)	.39				.81
16. I am angry about a lot of things.	0.6 (0.8)	.55				.68
11. I feel irritable.	1.3 (0.9)	.48				.59
13. I have a lot of regret about my life.	0.5 (0.8)	.51				.53
Items excluded from factor analysis due to extreme kurtoses						
2. My life seems to be pointless.	0.2 (0.6)	.53				
3. There is no purpose to the activities in my life.	0.2 (0.6)	.40				
14. Life in no longer worth living.	0.1 (0.3)	.40				
20. I would rather not be alive.	0.1 (0.3)	.41				

Only factor loading coefficients greater or equal to .40 are shown. Positively worded items (1, 6, 12, 17, 19) have been reverse scored

Depression symptoms were measured with the Patients Health Questionnaire 9 (PHQ-9) [25]. Total scores range from 0 to 27. Cutoff points of 5, 10, 15, and 20 represent mild, moderate, moderately severe and severe depression respectively [26]. The PHQ-9 has performed acceptably in detecting clinically-diagnosed Major Depressive Disorder in women in the postpartum period compared to clinical interview diagnosis [27, 28] and has been found to be highly concordant with the Edinburgh Postnatal Depression Scale (EPDS) [27, 29].

Anxiety symptoms were measured with the Generalised Anxiety Disorder 7 (GAD-7) questionnaire [30]. Total scores range from 0 to 21. Cutoff points of 5, 10, and 15 represent mild, moderate, and severe anxiety respectively. Although designed primarily to measure symptoms of generalised anxiety disorder, the GAD-7 has also demonstrated satisfactory performance as a screening measure for other anxiety disorders [31].

The personality vulnerability subscale of the Vulnerable Personality Style Questionnaire (VPSQ) [20] was used to measure personality characteristics, such as a tendency to worry, over-sensitivity to the opinions of others and lack of assertiveness, known to be associated with depression symptoms in the postnatal period. This subscale of the VPSQ consists of six items, with a maximum score of 30 indicating high personality vulnerability. The VPSQ also has another subscale consisting of three items, organisation/responsiveness, measuring general coping and emotional responsiveness. However, the organisation responsiveness subscale has been found to be less stable over time than the personality vulnerability scale and to have low internal reliability (Cohen's alpha of 0.18 to 0.36) [20]. In our study the organisation/responsiveness scale similarly had very low Cohen's alpha coefficient (0.20) and also reduced the internal reliability of the total VPSQ score (Cohen's alpha = 0.52 with both subscales included; Cohen's alpha = 0.70 for the vulnerability subscale only). Therefore, we only used the personality vulnerability subscale in the present study.

Psychiatric history was assessed by self-report, asking respondents whether prior to having the baby they had been diagnosed or treated for any of the following conditions: alcohol or drug dependence; depression; anxiety; eating disorders; post traumatic stress disorder, or any other major psychiatric conditions.

Self-rated general health was measured with a single question from the SF-36 asking respondents to rate their general health as excellent, very good, good, fair, or poor [32]. Responses to this question are associated with symptoms of physical functioning and wellbeing [33].

Six questions about feeding in the previous 24 h were used, based on the World Health Organisation [34] indicators for assessing breast feeding practices to establish whether participants were breast and/or formula feeding.

Infant crying and fussing was measured with a shortened version of the Barr Parental Diary [35], consisting of two questioned about the number of hours that the infant cried/fussed over the two 12 h periods in the previous 24 h.

The quality of the relationship with the woman's intimate partner was assessed using the Intimate Bonds Measure (IBM) [36], which has two subscales: Care (assessing sensitivity, warmth, emotional responsiveness, trust, physical gentleness and kindness) and Control (coercion, dominance, exertion of power and criticism). Each subscale consists of 12 items, with scores ranging from 0 to 36, with higher scores indicating a more caring or a more controlling relationship respectively.

### Analysis

To examine the internal reliability of the Demoralisation Scale, Cronbach's alpha coefficient was calculated. To examine the factor structure of the scale exploratory factor analysis was conducted. Discriminant validity was examined by estimating Pearson's correlation coefficients between the Demoralisation Scale and its subscales with the PHQ-9, GAD-7 and vulnerable personality characteristics. Further evidence of good discriminant validity would be shown by a group of women who are categorised as high on demoralisation but not on depression or anxiety. To investigate this, the sensitivity (proportion of women with moderate to severe depression or anxiety who also have high demoralisation) and the positive predictive value (PPV) (proportion of women who were high on demoralisation but with no or mild depression or anxiety) [37] of the Demoralisation Scale were calculated. Cohen's kappa coefficients were also estimated. Cohen's kappa [38] coefficients are used to measure the extent of agreement between raters or classification scales [39], compensating for chance agreement. In the absence of a validated cutoff point for the Demoralisation Scale, a cutoff of 30, the mean obtained in clinical samples [12, 13] was used. The PHQ-9 and GAD-7 were categorised based on their published cutoff scores for moderate depression and anxiety respectively (≥10) [26, 30].

To examine the unique association of demoralisation with various demographic and postnatal factors, independent of other measures of distress, hierarchical linear regression was carried out. In all correlational and regression analyses some variables (demoralisation, PHQ-9, GAD-7, and the IBM control subscale) were natural log transformed to correct for positive skewness and outliers. The IBM care subscale was first reflected (by subtracting each score from the highest score plus one) and then natural log transformed, to correct for negative skewness. Self-rated health was dichotomised as "fair to good" versus "very or extremely good", as it did not follow a normal distribution even after transformations. The number of hours of infant crying is a count variable and therefore it is inappropriate to apply a transformation to it [40]. It was dichotomised as <3 versus ≥3 h of crying during the day or night in the last 24 h, consistent with the widely used Wessel Criteria [41]. In our data three or more hours of crying was close to the 80th percentile. Analyses were conducted with IBM SPSS 20.0 [42].

## Results

### Participants

Six hundred and seventy eligible primiparous women were invited to participate, representing all potentially eligible women seen in the MCHCs during the time period of the study. Of the 670, 460 gave consent (68.7 %). Of the 460, 400 (87.0 %) completed the assessment interview from 1.1 to 15.6 weeks after birth (median = 5.5 weeks, interquartile range (IQR) = 3.9-7.7).

### Demographics

Participants' mean age was 31.0 years (SD = 5.1; median = 31.0; range = 18-43). Nearly all (95.8 %) were living with a partner, more than half (62.3 %) had completed tertiary education, 16.8 % were from a non-English speaking background. These demographic characteristics are reasonably comparable to available data of women giving birth in the state of Victoria, Australia: median age of primiparous women 30.0 years [43]; 86.9 % with a current partner; and 21.4 % of non-English speaking background [44].

### Postnatal demoralisation in the community

The mean of the Demoralisation Scale was 14.9 (SD = 12.0, range = 0-67) and the median was 12.0 (IQR 7–18). This indicates a low overall level of demoralisation among the respondents, compared to a mean of 30 in clinical samples postnatally [12] and in palliative care [13, 45]. Approximately 8 % of respondents in the present study scored 30 or above, indicating a relatively high level of demoralisation. As a comparison, scores on depression (M = 3.8; SD = 3.5; median = 2.0 IQR = 1.0-5.0 on the PHQ-9) and anxiety (M = 3.3; SD = 3.5; median = 2.0; IQR = 1.0-5.0 on the GAD-7) were in the mild range (<5) of the recommended cutoff points [26].

### Reliability of the Demoralisation Scale

The Demoralisation Scale had excellent internal consistency (Cronbach's alpha = 0.90). Corrected item-total correlations (Table 1) ranged from 0.27 to 0.68.

### Factor analysis of the Demoralisation Scale

Initially, factor analysis was attempted with all the items of the Demoralisation Scale. However, four items did not exhibit a meaningful pattern of factor loadings. These items were scored 0 by most respondents (87.3 %-97.3 %) and had extreme kurtoses [46] (ranging from 9.6 to 57.6). They were, therefore, unsuitable for factor analysis [46] and were excluded. These four items were part of the "Loss of Meaning" scale that has been extracted in a previous study of the Demoralisation Scale in a hospital mother-baby unit among women who had recently given birth [12] and with cancer patients [13, 45, 47]. Although the above items were excluded from the factor analysis, they were retained in the scale for the rest of the analyses, in order to be able to compare the total score of the Demoralisation Scale to that of previous studies. The reliability (Cohen's alpha) of the total scale remained the same with and without the above four items.

In the present study, factor analysis was conducted with the remaining 20 items, using the principal components extraction method and a Varimax rotation [48], with eigenvalues ≥1 and based on the scree plot, accounting for 54.1 % of the variance. An oblique rotation method had similar results. Only results of the Varimax rotation are reported here. As shown in Table 1, in the present study we extracted four factors: helplessness, disheartenment, sense of failure, and dysphoria. As these factors were conceptually similar to Kissane et al.'s [13] factors, we used the same names. Two items (21 and 18) with significant loadings greater than .40 on two factors were included in the factor with the higher loading. Item 6 ("I am in good spirits") loaded slightly higher on the sense of failure factor (.48) than the disheartenment factor (.43), although it was more conceptually consistent with the latter. Therefore, we included this item in the disheartenment factor. This was also more consistent with the factor structures obtained in previous studies [12, 13].

In comparison, Kissane et al. [13] extracted five factors in their validation study of the Demoralisation Scale among patients in palliative care: loss of meaning (items 14,20,2,4,3); dysphoria (items 15,16,10,11,13); disheartenment (items 12,24,22,23,6,21); helplessness (items 7,8,5,9) and sense of failure (17,1,12,19). In our previous study of postnatal demoralisation in a hospital mother-baby unit [12], we extracted four factors: a factor which was mainly a combination of the dysphoria and disheartenment items (items 11,16,15,21,10,18,5,9,22); helplessness (items 24, 23,7,4,8,6); loss of meaning (items 14,20,2,13,3); and sense of failure (items 17,19,1,12). The sense of failure factor consisted of identical items across all three studies. The dysphoria and disheartenment items were reasonably similar across all studies, although they loaded on the same factor in the Bobevski et al. [12] study. There was some variation in the items of the helplessness factor, although in all three studies items 7 ("no one can help me") and 8 ("I feel that I cannot help myself") loaded on this factor. Overall, the dimensions of demoralisation that emerged were consistent across the three studies, but not identical.

The internal consistency of the four factors ranged from acceptable to good (Cronbach's alpha coefficients 0.63 to 0.83) (Table 1). As the distributions of the total score and the factor subscales were positively skewed, each variable was natural log transformed in order to calculate the Pearson correlation coefficients between the subscales. The subscales were weakly to moderately correlated with each other and were moderately to highly correlated with the total score (Table 2).

**Table 2:** Correlation matrix of the Demoralisation Scale subscales and other measures distress related measures

	F1	F2	F3	F4	Demoralisation Scale	PHQ-9	GAD-7	VPS Personality vulnerability
F1: Helplessness^a^ (mean of items = 0.5; SD = 0.5)	-							
F2: Disheartenment^a^ (mean of items = 0.6; SD = 0.2)	.68*	-						
F3: Sense of Failure^a^ (mean of items = 1.0; SD = 0.3)	.33*	.45*	-					
F4: Dysphoria^a^ (mean of items = 0.9; SD = 0.4)	.58*	.53*	.33*	-				
Total demoralisation score^a^	.78*	.84*	.68*	.76*	-			
PHQ-9^a^ (α = .73)	.40*	.52*	.36*	.45*	.54*	-		
GAD-7^a^ (α = .85)	.48*	.55*	.31*	.48*	.56*	.68*	-	
VPS personality vulnerability (α = .70)	.40*	.35*	.31*	.45*	.47*	.47*	.47*	-

^a^Natural log transformed variables

*p > .0001

### Discriminant validity

#### Correlations between demoralisation and other measures of distress

Pearson's correlation coefficients between the Demoralisation Scale and other measures of distress are shown in Table 2. The Demoralisation Scale and its subscales correlated only moderately with the other measures of distress, thus showing evidence for good discriminant validity.

#### Comparison of categorical scores of the Demoralisation Scale with the PHQ-9 and GAD-7

Table 3 shows a good separation between the Demoralisation Scale and the PHQ-9 and GAD-7, with low proportions of the respondents with high demoralisation scores also having moderate to severe depression (32.0 %) or anxiety (38.7 %), as indicated by the PPVs. Sensitivity was also low (40 % with the PHQ-9; 52.2 % with the GAD-9). Cohen's kappa coefficients ranged from 0.25 to 0.41, indicating only fair to moderate overall agreement [49]. Overall, the above results indicate good discriminant validity between the Demoralisation Scale and the PHQ-9 and GAD-7.

**Table 3:** Comparison of categorical scores of the Demoralisation Scale with the PHQ-9 and GAD-7

Comparison Scale	Demoralisation Scale cutoff score	Sensitivity	Positive Predictive Value (PPV)	Cohen's kappa
PHQ-9 (cutoff score ≥ 10)	≥30	40.0 % (10/25)	32.0 % (10/31)	0.25
GAD-7 (cutoff score ≥ 10)	≥30	52.2 % (12/23)	38.7 % (12/31)	0.41

### Postnatal factors associated with demoralisation

The unique association of demoralisation with variables related to women's early postnatal experiences (mode of birth, unexpected pregnancy, mother's health, infant feeding, infant crying and fussing, confidence on going home from hospital after birth, and relationship with partner), after controlling for demographic and distress related variables, was investigated through hierarchical linear regression analysis. At first, univariate relationships between demoralisation and the above variables were investigated using separate simple linear regressions, with demoralisation as the dependent variable and each of the above variables as the independent variable. Variables that had a statistically significant (*p* < .05) univariate relationship with demoralisation were then entered into the hierarchical regression model, described below.

Table 4 shows the unadjusted unstandardised coefficients of the simple linear regressions. As the Demoralisation Scale scores were log-transformed in the regression analyses, geometric means and SDs of demoralisation are shown for the categorical independent variables, as well as the medians and IQRs for ease of interpretation. For the continuous independent variables, the correlation coefficients with the Demoralisation Scale are shown. Higher levels of demoralisation were significantly associated with non-English speaking background, lower educational level, psychiatric history prior to the birth, low health self-rating, formula feeding, infant's prolonged crying, high parental confidence, and perception of partner as being more caring and less controlling. There was also a trend approaching statistical significance (*p* = .055) for women aged 18–25 years to have slightly higher demoralisation compared to women aged 26–39 years.

**Table 4:** Percentage of participants with high demoralisation score by demographic and postnatal variables

		Geometric mean (SD) of the Demoralisation Scale	Median (IQR) of the Demoralisation Scale	Unstandardised regression coefficient (unadjusted)	*p*
Language	English (*n* = 333)	11.7 (2.1)	11.0 (7–18)	Reference	
	Other (*n* = 67)	16.0 (1.9)	16.0 (10–26)	0.32	.001
Education	Up to secondary (*n* = 74)	10.3 (2.1)	10.0 (7.0-19.0)	Reference	
	Diploma/Certificate (*n* = 77)	13.4 (2.0)	12.0 (8.0-19.5)	0.25	.008
	Tertiary (*n* = 249)	12.7 (2.0)	12.0 (5.8-16.0)	0.27	.019
Age	18-25 (*n* = 54)	11.0 (2.2)	10.0 (5.8-15.3)	−0.20	.055
	26-39 (*n* = 328)	12.6 (2.0)	12.0 (7.0-19.0)	Reference	
	40+ (*n* = 18)	12.0 (1.6)	12.0 (6.2-15.0)	−0.08	.632
Mode of birth	Vaginal spontaneous (*n* = 175)	12.0 (2.1)	12.0 (7.0-18.0)	Reference	
	Vaginal assisted (*n* = 87)	12.4 (1.9)	12.0 (5.0-18.0)	0.07	.481
	Caesarian without labour (*n* = 59)	13.6 (1.9)	13.0 (5.0-18.0)	0.15	.153
	Caesarian after a period of labour (*n* = 79)	12.3 (2.1)	13.0 (4.0-21.0)	0.05	.580
Unexpected pregnancy	No (*n* = 305)	12.0 (2.0)	12.0 (7.0-18.0)	Reference	
	Yes (*n* = 95)	13.6 (2.0)	13.0 (8.0-13.0)	0.12	.135
Self-rating of health	Fair to good (*n* = 64)	18.1 (2.0)	17.5 (10.3-27.8)	Reference	
	Very good to extremely good (*n* = 335)	11.5 (2.0)	11.0 (7.0-17.0)_	−0.45	.000
	Missing (*n* = 1)				
Feeding method	Breast milk only (*n* = 267)	11.8 (2.0)	11.0 (7.0-18.0)	Reference	
	Formula only or with breast milk (*n* = 118)	13.8 (2.2)	14.0 (8.8-23)	0.16	.042
	Missing (*n* = 15)				
Baby crying/fussing	<3 h (*n* = 293)	11.4 (2.1)	11.0 (6.0-17.0)	Reference	
	≥3 h (*n* = 107)	15.3 (1.8)	14.0 9.0 (14.0-21.0)	.027	.001
Psychiatric history	No (*n* = 302)	11.4 (2.0)	11.0 (7.0-17.0)	0.29	.000
	Yes (*n* = 98)	16.1 (2.0)	15.0 (9.0-23.3)		
			Correlation with the Demoralisation Scale: r (p)		
Confidence in going home from hospital			-.39 (*p* = .000)	−0.25	.000
IBM care subscale^a^			.22 (*p* = .000)	.018	.000
IBM control subscale^b^			.24 (*p* = .000)	.021	.000

The dependent variable, the Demoralisation Scale total score, has been natural log transformed in the regression analysis

^a^Reflected and natural log transformed in the regression analysis

^b^Natural log transformed in the regression analysis

To further investigate which postnatal experiences are independently related to demoralisation after the effect of demographics and overlapping distress variables are controlled for, a hierarchical multiple regression was carried out (Table 5). Demographic variables and the distress related measures (depression, anxiety, and personality vulnerability) were entered at the first stage (Model 1). The postnatal variables that had a statistically significant bivariate association with demoralisation (*p* < .05) were entered at the second stage (Model 2) to examine whether they were significantly and independently associated with demoralisation after demographics and other distress related measures were controlled for.

**Table 5:** Hierarchical regression with demoralisation as the dependent variable

	Hierarchical linear regression				
Independent Variables		Unstandardised regression coefficient (adjusted)	p	Model R^2^	p for change in R^2^
	Model 1			.44	-
Language spoken at home: English		Reference			
Other		0.21	.007		
Education: Up to secondary		Reference			
Certificate/Diploma		0.12	.119		
Tertiary		0.17	.058		
Age: 18-25		−0.15	.093		
26-39		Reference			
40+		0.04	.790		
Psychiatric history		0.09	.183		
PHQ-9^a^		0.27	.000		
GAD-7^a^		0.21	.000		
Vulnerable personality characteristics^a^		0.05	.000		
	Model 2			.51	.000
Language spoken at home: English		Reference			
Other		0.13	.097		
Education: Up to secondary		Reference			
Certificate/Diploma		0.08	.320		
Tertiary		0.14	.115		
Age: 18-25		−0.15	.078		
26-39		Reference			
40+		−0.00	.990		
Psychiatric history		0.08	.221		
PHQ-9^a^		0.21	.000		
GAD-7^a^		0.19	.000		
Vulnerable personality characteristics		0.04	.000		
Self-rated health: Fair to good		Reference			
Very or extremely good		−0.18	.018		
Feeding method: Only breast milk		Reference			
Formula and/or breast milk		−0.05	.377		
Baby crying or fussing: <3 h		Reference			
≥3 h		0.12	.046		
Confidence in going home from hospital		−0.13	.000		
IBM care subscale^b^		0.04	.236		
IBM control subscale^a^		0.10	.006		

The dependent variable, the Demoralisation Scale total score, has been natural log transformed

^a^Natural log transformed

^b^Reflected and natural log transformed

A total of 19 (4.8 %) respondents had missing data on either method of feeding, health self-rating, or the IBM. In the univariate analyses of these variables (Table 4) missing data was excluded. All available data from all 400 participants was included in the hierarchical regression presented in Table 5. As there is a minimal amount of missing data and as there was no statistical significant difference in demoralisation scores between respondents with missing and non-missing data, this approach appeared appropriate [46]. The hierarchical regression was also repeated with omitting the 19 respondents with missing data with nearly identical results (not presented here).

Table 5 shows that even after the demographic variables and the distress related measures were controlled for, some factors were significantly associated with higher demoralisation, with a significant increase in R^2^. Mother's health problems, infant crying or fussing for three or more hours, low confidence in going home from the hospital, and a controlling partner were significantly associated with higher levels of demoralisation. These results also provide further evidence for good discriminant validity of the construct of demoralisation, as they show that demoralisation is uniquely associated with adverse postnatal experiences, even after overlapping distress variables are controlled for.

## Discussion

### Key findings

The Demoralisation Scale was found to have good internal consistency and discriminant validity among women who had recently given birth to a first baby. Four subscales were identified: helplessness, disheartenment, sense of failure, and dysphoria. About 8 % of participants experienced high levels of demoralisation. High levels of demoralisation were independently associated with low confidence on going home from the hospital after birth, low self-rated global health, more than 3 h of infant crying or fussing in the last 24 h, and a controlling partner, even after demographics and overlapping distress variables were controlled for.

### Subscales of the Demoralisation Scale

The subscales that were extracted through factor analysis in the present study were consistent with, but not the same, as those extracted in a hospital mother-baby unit [12] and in palliative care settings [13]. The main difference was that in the present study loss of meaning did not appear to be a salient experience in the early postnatal period, with nearly all participants scoring zero on this group of items. Loss of meaning has been found to be an important aspect of demoralisation among people with cancer [13, 45, 47]. A loss of meaning factor was also extracted in a previous study of women admitted to a hospital mother-baby unit [12], although it had the lowest item mean scores compared to the other scales. Thus, experiences of loss of meaning appear to be uncommon among women in the community during the early postnatal period. However, it appears to be an aspect of the more severe levels of demoralisation found among women admitted to clinical services. The relatively low salience of loss of meaning postnatally is not surprising [12], as even women with predominantly negative postnatal experiences report co-existing positive feelings about motherhood, such as watching the baby's development and feeling needed and loved [50].

### Discriminant validity

Of the participants who scored high on demoralisation, about 68 % and 61 % did not report substantial symptoms of depression or anxiety, respectively. There was poor agreement of classifications between the Demoralisation Scale and the PHQ-9 and GAD-7, as indicated by small Kappa coefficients. Furthermore, demoralisation was independently associated with a number of postnatal experiences. This suggests that the Demoralisation Scale has good discriminant validity. Our previous study of demoralisation in a hospital mother-baby unit [12] also found evidence for acceptable discriminant validity of the Demoralisation Scale. In comparison, depression is a broader, heterogeneous construct, including anhedonia, as well as somatic symptoms, such as sleep and appetite problems. It is commonly argued that in assessing depressive disorders in the postnatal period less emphasis should be put on somatic symptoms, as they are often normal, but the loss of pleasure and increased irritability are important features [51]. Neither the available dimensional measures of depression nor the psychiatric diagnostic systems specifically address helplessness and subjective incompetence. Many qualitative studies of women's own accounts, however, have consistently found that feelings of helplessness and a sense of failure are salient features of the postnatal period [50, 52, 53].

### Prevalence of symptoms of demoralisation

About 8 % of participants obtained high demoralisation scores, defined as above the mean of the Demoralisation Scale in clinical settings (score of ≥30) [12, 13, 45]. A total of 5 % of all participants scored 30 or above on demoralisation without substantial depressive symptoms. In the absence of normative demoralisation scores, this can be tentatively considered as an indication of prevalence of symptoms of high demoralisation among primiparous women in the community in the early postnatal period. This is a non-trivial prevalence that is similar to that of depressive (5.1 %) and anxiety (2.2 % to 8.3 %) disorders among women in the Australian population [54].

### Factors associated with demoralisation

Women's circumstances in the early postnatal period, such as their health, infant behaviour, and relationship with their partner were uniquely associated with high levels of demoralisation. This suggests that stressful postnatal circumstances are related to experiences of distress, helplessness and sense of failure that are not sufficiently accounted for by the traditional focus on symptoms of depressive and anxiety disorders. Although the measures of depression and anxiety symptoms and of personality vulnerability together explained a large proportion of the total variance in the regression model, this is to be expected as distress-related variables overlap and are usually strongly related to each other.

Many of our findings were consistent with previous studies on psychological distress in the postnatal period. Unsettled infant behaviour has been associated with symptoms of depression [55, 56], anxiety and parental stress [57]. Intervention programs focusing on assisting women to manage unsettled infants also decrease symptoms of depression and anxiety [58–60], as well as demoralisation [12]. The mother's ill health and a lack of supportive and caring relationship or a coercive and controlling partner have been associated with high psychological distress postnatally [17]. Parental confidence or self-efficacy is an important factor in maternal mental health [61]. Anxiety about infant care on discharge from hospital following child birth has been associated with high psychological distress in the early postnatal period [17].

We found a statistically significant univariate relationship between higher demoralisation and formula only or formula and breast feeding, compared to exclusive breast feeding. However, this relationship was no longer significant once other factors were controlled for. A recent systematic review of breastfeeding and depression [62] found a clear link between early exclusive and non-exclusive cessation of breastfeeding and postnatal depressive symptoms, with prospective evidence that depression often precedes and leads to breast feeding cessation. However, there is also evidence that negative breast feeding experiences can precede the onset of depressive symptoms [62]. Further investigation of the relationship between demoralisation and breastfeeding from prospective studies is warranted.

Previous studies have found lower educational attainment and younger (18–25 years) and older (40+ years) age to be a risk factor for postnatal depressive symptoms compared to 26–39 years of age (e.g. [63]). We did not find the same pattern of relationships with demoralisation. In fact, in the present study there was a statistically significant univariate relationship between higher demoralisation and higher educational attainment, as well as a trend for slightly higher demoralisation among participants in the middle age band compared to younger participants. Although once we controlled for other factors these relationships were not statistically significant, these trends also warrant further investigation in future studies. The Demoralisation Scale contains questions about loss of role and a sense of incompetence that are not part of standard anxiety and depression measures but may be especially relevant to the early postnatal period. Women with higher educational attainment and aged 26–39 years may, for instance, still be getting established in their career, and following the birth of a baby may feel that they have lost a previous role in life at which they felt competent, but that their previous life experiences have not prepared them for the new role of caring for an infant. Pervasive loss of previous roles, autonomy and control is a major recurring factor in qualitative accounts of women's postnatal experiences [53, 64].

### Implications

Our earlier study of demoralisation in a hospital mother-baby unit [12] found that an intervention program that focused on psychoeducation and skill-building resulted in more participants improving significantly on demoralisation than on depression and anxiety symptoms. Thus, recognition of the salience of demoralisation could direct population based programs to help women acquire care giving skills and increase their parental self-efficacy in order to decrease feelings of helplessness and sense of incompetence. The use of the demoralisation construct in population based mental health programs may also reduce stigma, which is still a major barrier to help seeking in the postnatal period [65, 66]. If practitioners communicate and intervene in a way that is more meaningful and relevant, perhaps women will feel that their experiences are better recognised and less stigmatised, and will be more prepared to seek help.

Slavney [67] has argued that demoralisation is part of a normal adjustment process, whereas most other authors [68, 69] view it as a more serious form of distress that is clinically relevant and deserves both recognition and treatment. As demoralisation is a dimensional phenomenon, at the mild end it may constitute a normal reaction to stress, but with increasing distress and increasing subjective incompetence it could become more serious and require intervention [69]. In patients with physical illness demoralisation is of clinical significance because it is associated with the desire to die [11]. In postnatal settings, our previous study [12] found that demoralisation was uniquely associated with negative experiences of motherhood and high impairment in functioning of women who had recently given birth, as evidence that high levels of demoralisation are of clinical importance postnatally.

Even in the absence of depression and anxiety, women can feel they have lost their sense of competence, feel helpless, and distressed. A sense of incompetence as a caregiver has been identified as a risk factor in the development of maternal mental health problems [61, 70] and has been linked to infant developmental outcomes [61]. If symptoms of demoralisation persist or become more severe and help is not available, feelings of hopelessness can increase too [68]. Hopelessness is a well established predictor of suicidal ideation and intent, independently of depression [71–73]. Our previous study showed that demoralisation can be substantially decreased by an intervention program based on parental skill building and psychoeducation [12]. It is, therefore, important for practitioners in postnatal clinical settings to identify women with high levels of demoralisation, even without concurrent depression or anxiety symptoms, and to offer them effective interventions in order to prevent serious long term problems for the mother and infant.

### Strengths and limitations

A strength of this study is that it used a large community sample that was recruited systematically from across the socioeconomic spectrum. The response rate (68.7 %) was reasonable. However, no information was available for non-responders and there may have been response bias. There were a relatively large proportion of participants with post secondary education (62 %) and there were very few participants without a current partner (4 %). The study is cross-sectional and, therefore, direction of relationships among the investigated variables cannot be inferred. Although this study used baseline data from a cluster randomised control trial of a postnatal intervention program, the baseline data was collected before the intervention was carried out and both the participants and the researchers were blinded to allocations to the control or intervention groups.

## Conclusions

The Demoralisation Scale is a reliable and valid tool for measuring demoralisation in the community among women who had recently given birth. Higher levels of demoralisation were independently associated with lower confidence about going home from the hospital after birth, lower rating of mother's self-rated global health, more than three hours of infant crying and fussing in the last 24 h, and a controlling partner, even after symptoms of depression and anxiety, and vulnerable personality characteristics were controlled for. The relevance of demoralisation to postnatal health practitioners in the community is in enabling them to better understand women's experiences and to intervene in a way that is more meaningful and less stigmatising to women, rather than exclusively focusing on reducing symptoms of depression. In population based postnatal programs this would bring the focus on helping first time mothers to acquire the necessary caregiving skills and to increase their parental self-efficacy, thereby decreasing their sense of helplessness and subjective incompetence.

## Abbreviations

DSM-5: Diagnostic and Statistical Manual of Mental Disorders, 5th Edition
WWWT: What Were We Thinking programme
CATI: Computer assisted telephone interview
MCHC: Maternal and Child Health Centre
LGA: Local government area
PHQ-9: Patients' Health Questionnaire 9
GAD-7: Generalised anxiety disorder 7
IBM: Intimate Bonds Measure
IQR: Interquartile range
